# Different Treatment Modality in the Management of Acute Mesenteric Ischemia

**DOI:** 10.7759/cureus.12490

**Published:** 2021-01-05

**Authors:** Mohamed Selim, Mona I Alnaimi, Zainab Y Alsadah, Ghadah A AlHarbi, Mohamed Nasr

**Affiliations:** 1 Vascular Surgery, Al Azhar Faculty of Medicine, Cairo, EGY; 2 Vascular Surgery, King Fahad University Hospital, Dammam, SAU; 3 Medicine, King Fahad University Hospital, Khobar, SAU; 4 Emergency Medicine, King Fahad University Hospital, Khobar, SAU; 5 Ophthalmology, King Fahad University Hospital, Khobar, SAU; 6 Vascular Surgery, King Fahad University Hospital, Khobar, SAU

**Keywords:** acute mesenteric ischemia, illio-mesenteric artery bypass, endovascular treatment, open approach.

## Abstract

Mesenteric ischemia (MI) is a reduction in blood flow of the mesenteric vessels resulting in ischemia or infarction if not treated properly. It is difficult to diagnose and it has a high rate of mortality. Moreover, it is managed either by open surgery or endovascular approach. We present a case of a 79-year-old male patient with MI managed initially by thrombolytic therapy with stent through inferior mesenteric artery which has failed. Few days later he presented with the same complaint. He was treated with heparin for five days and discharged in good condition. Six days later, the patient returned to the ED with MI and managed successfully with retrograde right common ilio-mesenteric artery bypass with no complication and made full recovery. Endovascular revascularization is a minimally invasive approach and it is the initial treatment of choice for mesenteric occlusive disease. However, this approach is not always feasible which explains the role for open approach.

## Introduction

Mesenteric ischemia (MI) is an inadequate blood flow through the mesenteric vessels resulting in ischemia or infarction if not treated properly. It is difficult to diagnose such case due to initial nonspecific symptoms before peritonitis presents. High rate of mortality reaching 30%-65% was reported in several studies. It is classified as either arterial or venous disease. Arterial MI can be subdivided into occlusive and non-occlusive [1-3].

The most common cause of MI is atherosclerosis. Less frequently encountered causes include dissection, trauma, mesenteric aneurysm rupture, and arteritis or fibromuscular dysplasia [3-4].

Mesenteric ischemia is a common cause of acute abdomen in elderly, especially in those with predisposing factors such as arrhythmias, congestive heart failure, diuretics, or digoxin. About 10% of elderly above 70 years of age with acute abdomen had acute MI. High index of suspicion will lead to early recognition and treatment which will reduce morbidity and mortality [1].

## Case presentation

A 79-year-old male patient presented to the ED complaining of abdominal pain, nausea, and vomiting. Medical history includes type-2 diabetes mellitus, hypertension, dyslipidemia, atrial fibrillation, and aortic dissection. Additionally, surgical history was remarkable for thoracic endovascular aortic repair (TEVAR) that was done five years ago.

**Figure 1:**
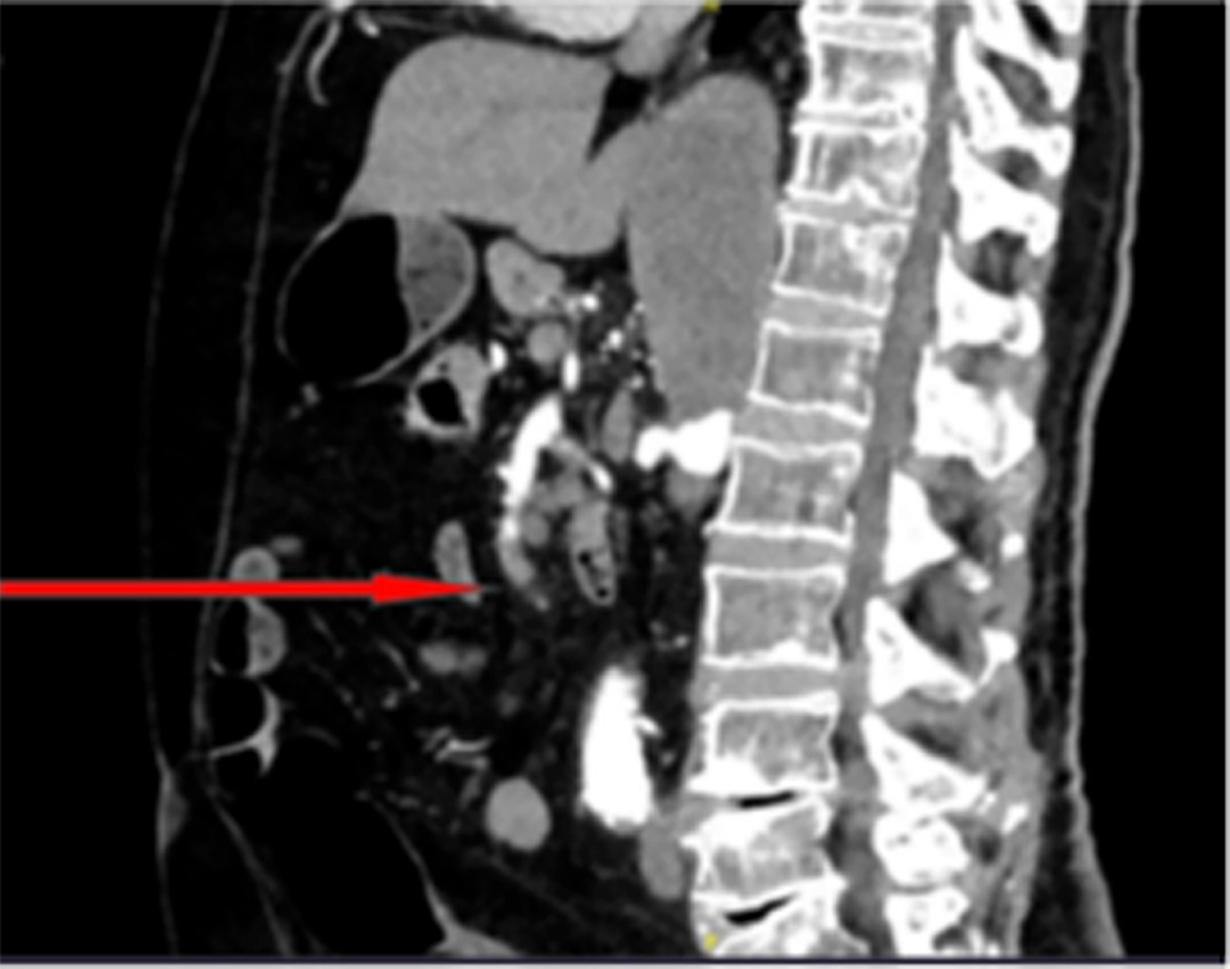
CT with contrast showing a distal occlusion of SMA. SMA, superior mesenteric artery

**Figure 2:**
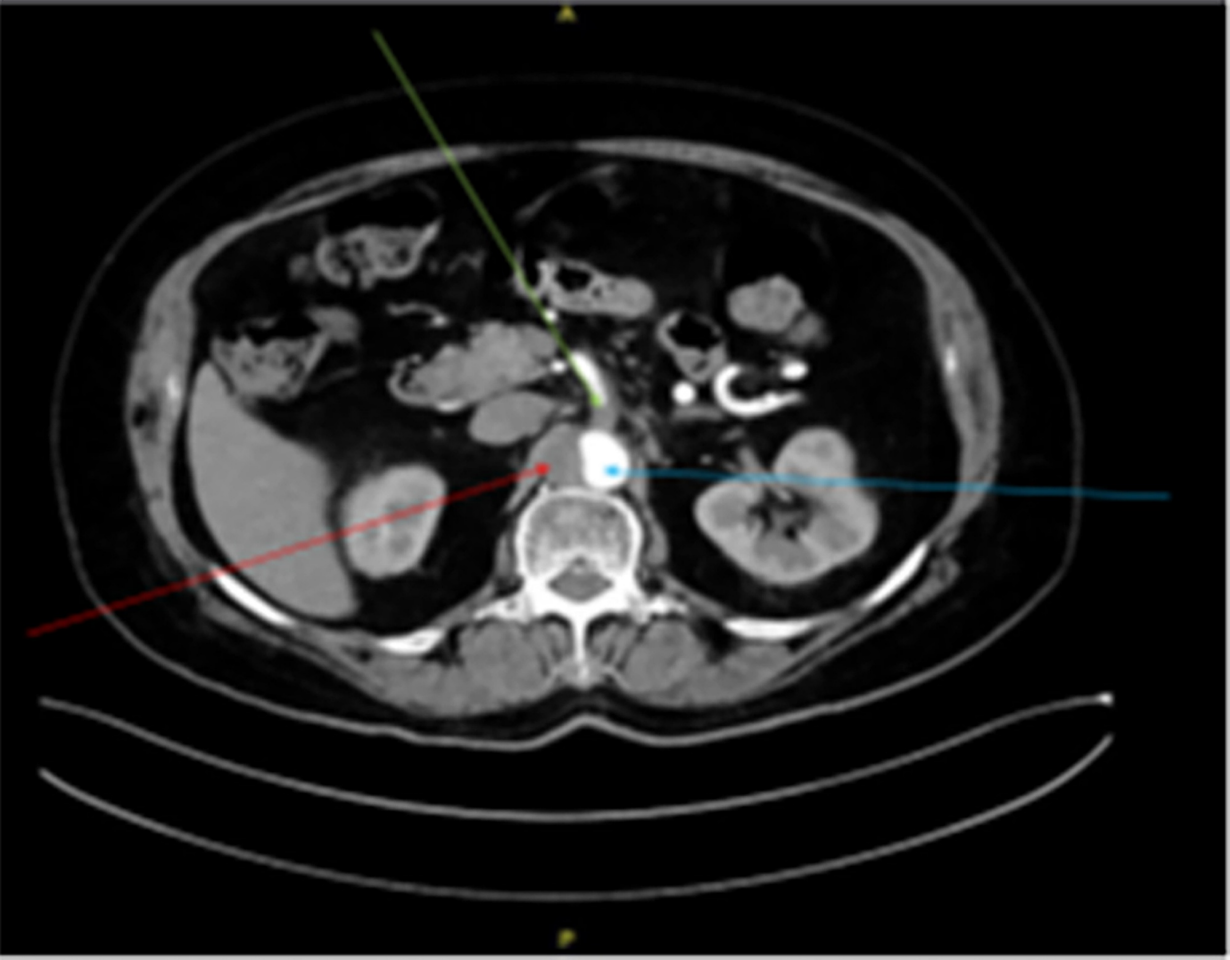
CT with contrast green arrow shows chronic occlusion of SMA, blue arrow shows true lumen of aorta, red arrow shows thrombus in aorta. SMA, superior mesenteric artery

During physical examination, the patient was found to be hemodynamically stable and his abdomen was soft and lax with mild tenderness on the left flank. Laboratory tests showed significant elevation of lactic acid (4.16 mmol/L) and white blood cells (WBC) (13.4 K/uL) with neutrophils 80.8%. CT angiography showed occlusive thrombus at distal superior mesenteric artery (SMA) (measuring about 2 cm) and obliterated origin of both celiac trunk and SMA with collateral from inferior mesenteric artery (Figure 1). In addition, thrombus at the proximal part of the abdominal aorta (Figure 2). 

The patient was admitted as a case of superior mesenteric artery (SMA) ischemia, and managed by thrombolytic therapy using tissue plasminogen activator (tPA) (alteplase 0.05 mg/kg/h for 12 hours) with stent on the same day through inferior mesenteric artery. There were no complications from the procedure. However, SMA stenting failed. The patient was improving and was discharged home on apixaban 2.5 mg.

Four days later, the patient presented to the ED with diffused sharp abdominal pain. The physical examination is unremarkable except for a mild central abdominal tenderness. Laboratory findings revealed lactic acid to be 2.19 mmol/L. CT angiography showed re-demonstration of near occlusion thrombus at distal SMA with a refill of distal branches from collaterals at the portal venous phase with no frank signs of bowel ischemia. The patient stayed in a regular ward and received heparin 1200 U/h; five days later the patient recovered and was discharged in good condition. 

**Figure 3:**
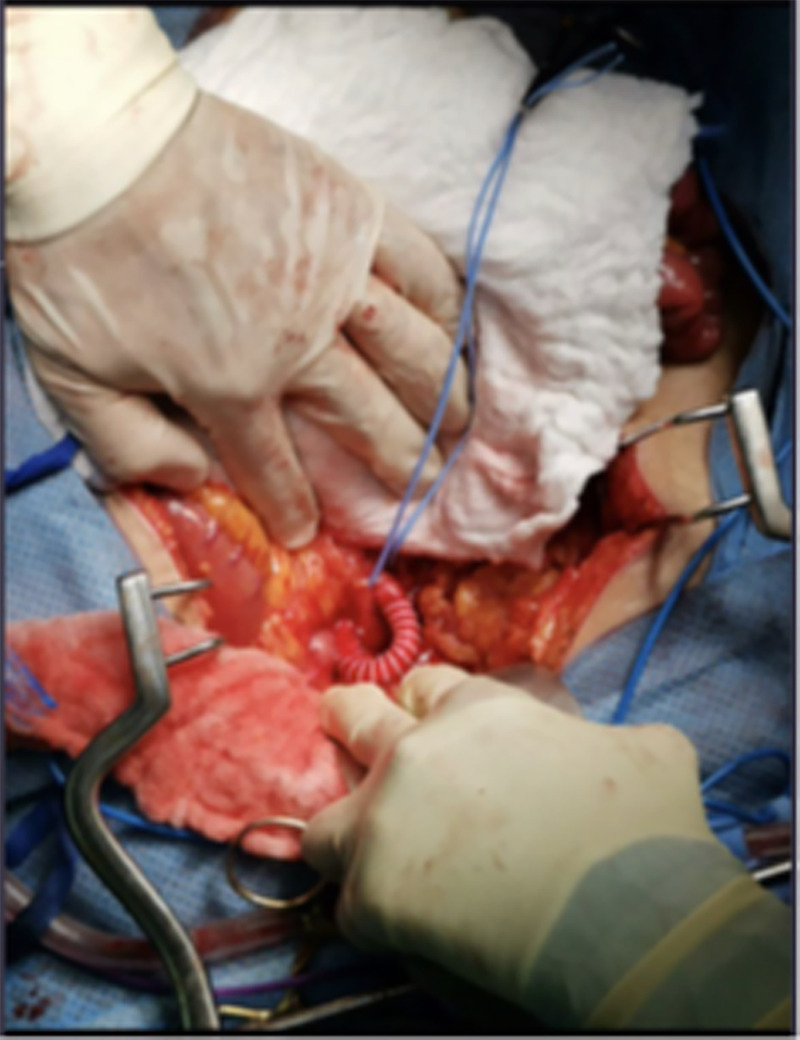
Intraoperative picture showing anastomosis of SMA with PTFE 8 mL externally supported. SMA, superior mesenteric artery; PTFE, polytetrafluoroethylene

Six days later, the patient returned to the ED with the same complaint and diagnosed with MI; he was vitally stable and afebrile; the abdomen is soft and lax with no tenderness. Laboratory tests revealed high lactic acid (2.94 mmol/L), lipase (48 U/L), and erythrocyte sedimentation rate (ESR) (28 mm/h). Angiography confirmed the presence of occlusive thrombus at distal SMA. The patient underwent a retrograde right common ilio-mesenteric artery bypass (Figure 3). He made an uneventful recovery and was discharged in a good condition.

## Discussion

The MI is a relatively rare but life threatening condition with high mortality rates reaching 30%-65% which reflect the importance of early intervention.

Management of acute mesenteric ischemia (AMI) is either through open surgery or endovascular approach. Choice of intervention depends on different factors, such as preoperative comorbidity, nutritional status, and anatomical stability. In general, endovascular intervention is preferred over open approach in selected cases when (1) no suspension of peritonitis or intestinal necrosis; (2) main trunk of SMA is not tortuous or angular; (3) proximal and distal landing zone are more than 10 mm and no main branches located in proximal and distal landing zone. Endovascular approach through groin or brachial access is preferred, given the possibilities to revascularize the branches and main trunk and the better results as reported in the literature. However, bias may present in endovascular approach cases by selecting the most fit patient for endovascular approach while advanced disease for open approach [2].

Endovascular intervention is the initial treatment of choice for SMA occlusion. However, this approach may not be feasible which justify the important role of the open surgical approach. An open approach is indicated in case of long segment occlusion in which a wire cannot be passed, calcified vessels, and failed angioplasty [5-6]. In this case, the patient presented with recurrent diffuse abdominal pain due to AMI despite trial of endovascular intervention. Initially the presence of thrombus at distal part of SMA due to atherosclerotic changes did not permit the wire to pass through the vessels itself so we tried to use endovascular thrombolytic and stenting indirectly through the inferior mesenteric artery branches, which was unsuccessful. As a consequence, we opted the last resort which is the open approach. 

There are several open surgical techniques for revascularization of the mesenteric artery which include the following: endarterectomy, bypass grafting, and mesenteric reimplantation. Mesenteric artery bypass constructs graft using two types of conduit either autologous reversed saphenous vein or prosthetic graft. The graft is either antegrade where the inflow is from the supraceliac aorta or retrograde bypass which is from the infrarenal aorta or iliac arteries [6]. Moreover, for our patient we decided to do a retrograde bypass because he has TEVAR and the presence of mural thrombus in the aorta.

## Conclusions

Mesenteric ischemia is relatively rare with high risk of mortality. Therefore, early diagnosis and prompt treatment is required to reduce morbidity and mortality. In addition, high suspicion in elderly present with an acute abdomen is required. Endovascular intervention is the initial treatment of choice for management of SMA occlusion. However, it is not feasible in all cases which justifies the important role of open surgical approach as in this case.
